# Enhancing Damage-Sensing Capacity of Strain-Hardening Macro-Steel Fiber-Reinforced Concrete by Adding Low Amount of Discrete Carbons

**DOI:** 10.3390/ma12060938

**Published:** 2019-03-21

**Authors:** Duy-Liem Nguyen, Dong-Joo Kim, Duc-Kien Thai

**Affiliations:** 1 Lecturer, Faculty of Civil Engineering, Ho Chi Minh City University of Technology and Education, 01 Vo Van Ngan St, Thu Duc District, Ho Chi Minh City 700000, Vietnam; 2 Department of Civil and Environmental Engineering, Sejong University, 209 Neungdong-ro, Gwangjin-gu, Seoul 05006, Korea; djkim75@sejong.ac.kr (D.-J.K.); thaiduckien@sejong.ac.kr (D.-K.T.)

**Keywords:** smart materials, electro-mechanical, resistivity, damage-sensing, hybrid fiber

## Abstract

The effects of adding micro-carbon fibers on the electro-mechanical response of macro-steel fiber-reinforced concretes (MSFRCs) under tension were investigated. Two MSFRCs were investigated and they had identical mortar matrix but different fiber contents: MSFRC1 and MSFRC2 contained 1.0 and 1.5 vol.% fibers, respectively. The volume contents of added micro-carbon fibers were 0 to 1.5 vol.% in MSFRC1 and 0 to 0.75 vol.% in MSFRC2, respectively. The addition of 0.5 vol.% micro-carbon fibers, in both MSFRC1 and MSFRC2, produced significantly enhanced damage-sensing capability and still retained their strain-hardening performance together with multiple micro cracks. However, when the content of carbon fibers was more than 0.5 vol.%, the MSFRCs generated tensile strain-softening behavior and reduced damage-sensing capability. Furthermore, the effects of temperature and humidity on the electrical resistivity of MSFRCs were investigated, as were the effects of adding multi-walled carbon nanotubes on the damage-sensing capability of MSFRCs.

## 1. Introduction

Structural health monitoring (SHM) has been frequently applied to monitor and inspect the structural performance of buildings and civil infrastructure during their long-term service [1,2]. Current SHM methods generally have utilized embedded or attached sensors; however, those sensors have high cost, low sensitivity and low durability [3]. In this study, we propose using self-sensing construction materials, i.e., materials that can sense strain/stress and damage/cracks by measuring the electrical resistivity of these materials under external loads. This approach has recently attracted much interest from many researchers because the proposed approach can overcome the disadvantages of using attached or embedded sensors. Self-sensing materials have been categorized as multifunctional or smart materials [4].

The self-sensing abilities of work-softening cement-based materials containing electrically conductive fibers have been intensively investigated since the 1990s [5,6]. Chen and Chung [5] reported that concrete containing 0.2 to 0.5 vol.% carbon fibers could sense elastic or inelastic deformation and fracture. Next, they also found the self-sensing ability of both mortar containing 0.2–4.2 vol.% carbon fiber and concrete containing 0.2–1.1 vol.% of carbon fiber [6]. Chung [7] and Wen et al. [8] also explored the self-sensing capacity of conductive fiber-reinforced cement-based materials. Although many previous studies have investigated the self-sensing response of work-softening cement-based materials, recent studies have focused on the self–sensing response of work-hardening cement-based materials. Macro-steel fiber-reinforced concretes (MSFRCs), with suitable fiber type and volume fraction, would have superior material properties such as high compressive strength, high tensile and bending strength, large ductility and energy absorption capacity under strain-hardening performance [9,10,11,12,13]. The unique tensile strain-hardening response accompanied by multiple micro cracks of MSFRCs is achieved by steel fiber bridging. It is really a superior mechanism to prevent catastrophic collapse as well as to enhance robustness, toughness and durability of the infrastructure. In addition to foregoing characters, the self-damage-sensing capability of MSFRCs has been discovered, i.e., there was a high relationship between the relative change in electrical resistivity and that in strain under tension [14,15,16]. The steel fiber-reinforced cementitious composites with various steel fiber types or contents produced strain-hardening behaviors and high damage-sensing capacities, compared with those of commercially conventional sensors (Nguyen et al. [14], Song at al. [15]). Lately, Kim et al. [16] also explored the damage-sensing ability of ultra-high-performance steel fiber-reinforced concrete. The multifunctional properties of MSFRCs are expected to increase their practical application. It is noticed that the damage-sensing property of strain-hardening composites was produced not only from conductive fibers but also from non-conductive fibers, i.e., both strain-hardening engineered cementitious composites (ECCs) and carbon black engineered cementitious composites (CB-ECCs) using PVA fibers demonstrated damage-sensing abilities, investigated by Li et al. [17] and Ranade et al. [18], respectively.

However, there is a great demand to enhance damage-sensing capacities of MSFRCs with low fiber content because the high volume content of fibers would generate difficulty in mixing, reduce their workability and increase the cost of MSFRCs. In this study, an effort to enhance the self damage-sensing capability of MSFRCs was conducted by adding micro-carbon fibers (CFs) or carbon nanotubes (CNTs) into them. Both CFs and CNTs are well-known as conductive materials with very high aspect ratio and high tensile strength. The synergy between macro-steel fiber and carbon additives is hoped to generate a favorable effect on electro-mechanical behaviors of MSFRCs. Besides, the electrical resistivity of MSFRCs, a key property that influences their damage-sensing capability, should be also investigated.

This situation has motivated the authors to perform this study, which is based on some partial previous reports [19,20,21] from the first author. The study is aimed to enhance the damage-sensing capability of MSFRCs, while still maintaining their strain-hardening behavior. The objectives of this study can be listed as follows: (1) to explore the influences of various additive types on the electrical resistivity of MSFRCs, (2) to explore the effects of humidity and temperature on electrical resistivity of MSFRCs, (3) to investigate the influences of carbon fiber volume fraction on the electro-tensile behavior of MSFRCs, (4) to review the damage-sensing capabilities of some MSFRCs, and (5) to investigate the effects of aligned multi-walled carbon nanotubes (MWCNTs) on the electro-tensile behavior of MSFRCs.

## 2. Experimental Test

**Figure 1:**
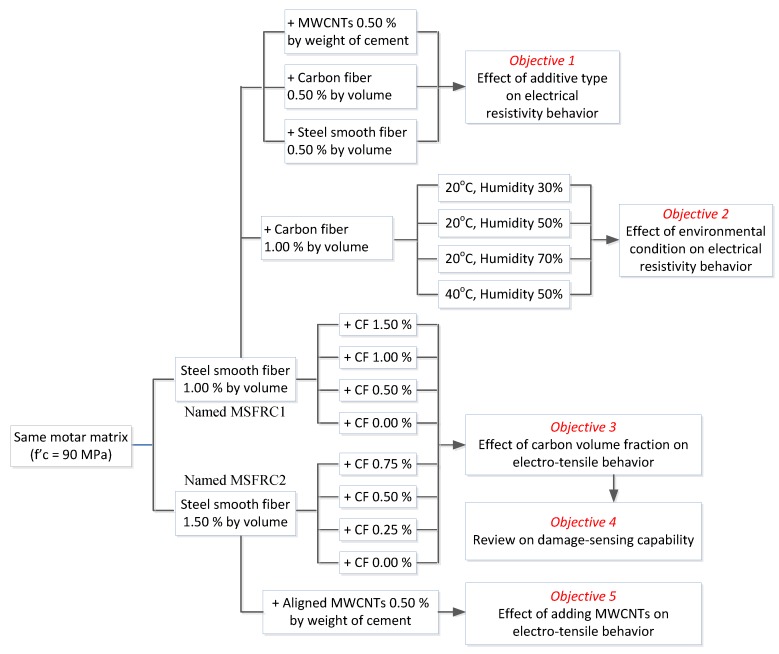
Experimental program on a flowchart.

**Figure 2:**
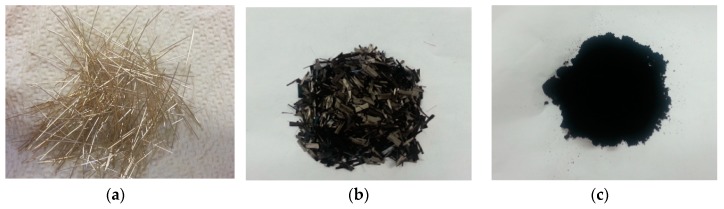
Conductive additives affixed into mortar matrix: (**a**) steel smooth fiber; (**b**) carbon fiber; (**c**) MWCNTs powder.

The experimental program and test series were designed to investigate the electrical resistivity and electro-tensile behaviors of MSFRCs, as summarized in the flowchart shown in Figure 1. All tested specimens used the same mortar matrix but with different conductive fibers or conductive particles added. For the first objective, the additives in specimens were as follows: (a) no conductive fiber nor powder, (b) 1.5 vol.% macro-steel smooth fibers, (c) 1.0 vol.% macro-steel smooth fibers blended 0.5 vol.% micro-carbon fibers, and (d) 1.0 vol.% macro-steel smooth fibers blended with 0.5% MWCNTs by weight content of cement. For the second objective, the additives included 1.0 vol.% macro-steel smooth fibers blended with 1.0 vol.% micro-carbon fibers. To investigate objectives 3 and 4, the tested specimens were prepared from two main MSFRCs containing different volume contents of macro-steel smooth fiber: MSFRC1 containing 1.0% and MSFRC2 containing 1.5%. For objective 3, the additional volume contents of carbon fibers in MSFRCs were changed: 0% (LS1.0-CF0.0), 0.5% (LS1.0-CF0.5), 1.0% (LS1.0-CF1.0) and 1.5% (LS1.0-CF1.5) for MSFRC1, and 0% (LS1.5-CF0.0), 0.25% (LS1.5-CF0.25), 0.50% (LS1.5-CF0.5) and 0.75% (LS1.5-CF0.75) for MSFRC2. For objective 5, the additional MWCNTs in MSFRC1 and MSFRC2 were unchanged, 0.5% by weight of cement. Figure 2 shows the photos of conductive fibers and powders used.

### 2.1. Materials and Preparating Specimens

All investigated MSFRCs were produced from an identical mortar matrix but different fiber contents. The mortar matrix included cement (type 3), silica fume, silica sand, fly ash, water and superplasticizer. Table 1 provides the composition of the mortar matrix and its compressive strength, while Table 2 and Table 3 provide the properties of macro-steel smooth fibers, micro-carbon fibers and multi-walled carbon nanotubes. Fine silica sand with grain diameters ranging between 0.15 and 0.7 mm was used, while cement, silica fume, and fly ash had a fineness of 4450, 163000, and 3637 cm^2^/g, respectively and a density of 3.15, 2.24, and 2.32 g/cm^3^, respectively. The compressive strength of the plain mortar matrix was 90 MPa. As given in Table 2, the steel smooth fibers were 30 mm long with a diameter of 0.3 mm, while carbon fibers were 12 mm long with a diameter of 7.2 μm. The tensile strength and elastic modulus of carbon fibers were 4137 MPa and 240 GPa, respectively, while those of steel fibers were 2580 MPa and 200 GPa, respectively. The carbon fibers had a density of 1.81 g/cm^3^ and an electrical resistivity of 1.2 kΩ-cm, whereas the steel fibers had a density of 7.9 g/cm^3^ and electrical resistivity of 2.06 × 10^−8^ kΩ-cm. As provided in Table 3, investigated MWCNTs had a length of 30–40 µm and a diameter of 10–15 nm. The MWCNTs included 10–15 walls with a density of 0.065 g/cm^3^ and a purity more than 90. The surface area of the MWCNTs was about 1200 m^2^/g.

**Figure 3:**
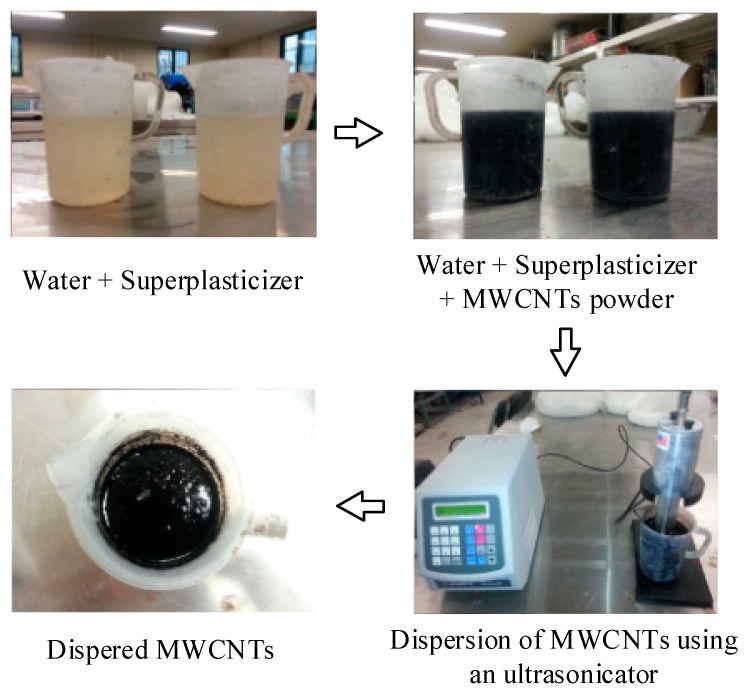
Schematic of MWCNTs dispersion.

All partial materials were mixed by a Hobart type mixer (Model KH-201, Troy, Ohio, USA) with a volume capacity of 20 L. Cement, sand, silica fume and fly ash was first dry-mixed for about 10 min. Then, water was added and further mixed for about 5 min. For the mixture containing MWCNTS, MWCNTs were pre-mixed with water by using a sonicator (Model VCX500, Sonics & Materials, Inc., Newtown, CT, USA) as can be seen in Figure 3. Superplasticizer was added to the mortar mixture after adding water and then further mixing. Steel fibers were carefully distributed into the mortar mixture when the mixture showed appropriate workability and viscosity for uniform fiber distribution, whereas carbon fibers were added with cement, sand, silica fume and fly ash prior to the addition of water. The mortar mixture was placed into molds using a wide scoop. All specimens were slightly vibrated to minimize the air bubbles inside them. After casting, the specimens were covered with plastic sheets. The specimens were placed in a laboratory at room temperature for 2 days prior to demolding. All specimens were placed in water for 14 days after demolding at 25 °C. The curing condition of high-performance fiber-reinforced concrete in this study was the same as those in reported studies [9,10,11,12,13,14,15,16,19,20,21]. The specimens after curing were dried for 12 h at 70 °C in an oven. The specimens were tested at the age of 18 days.

### 2.2. Test Setup and Procedure

**Figure 4:**
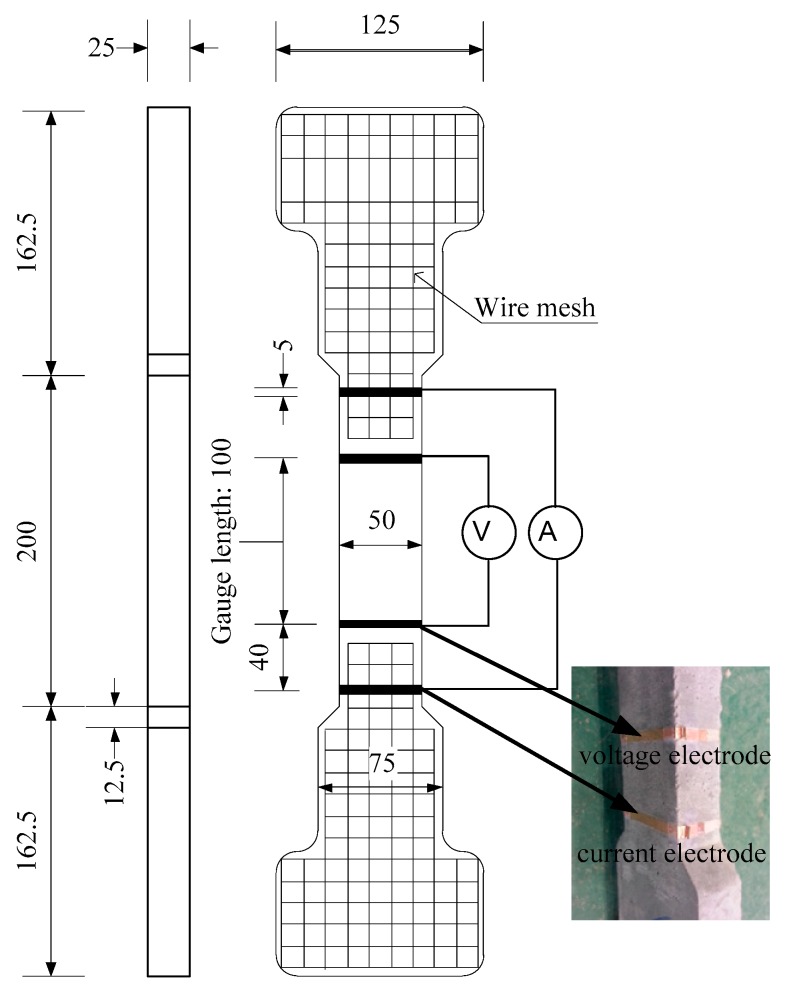
Specimen geometry.

**Figure 5:**
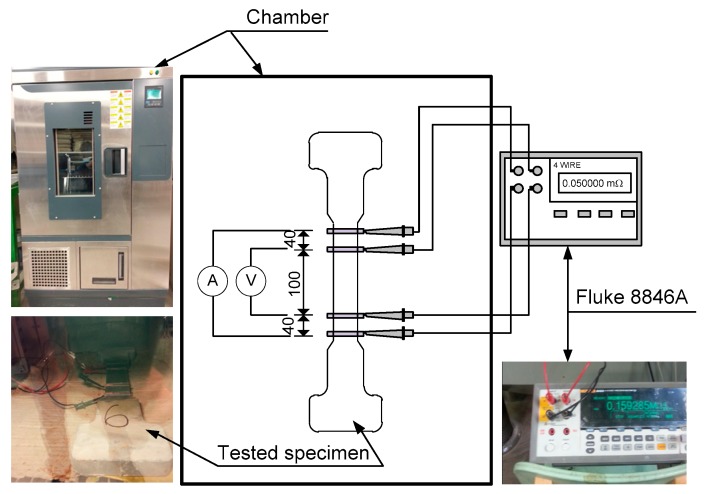
Test setup using a chamber to measure specimen resistance.

**Figure 6:**
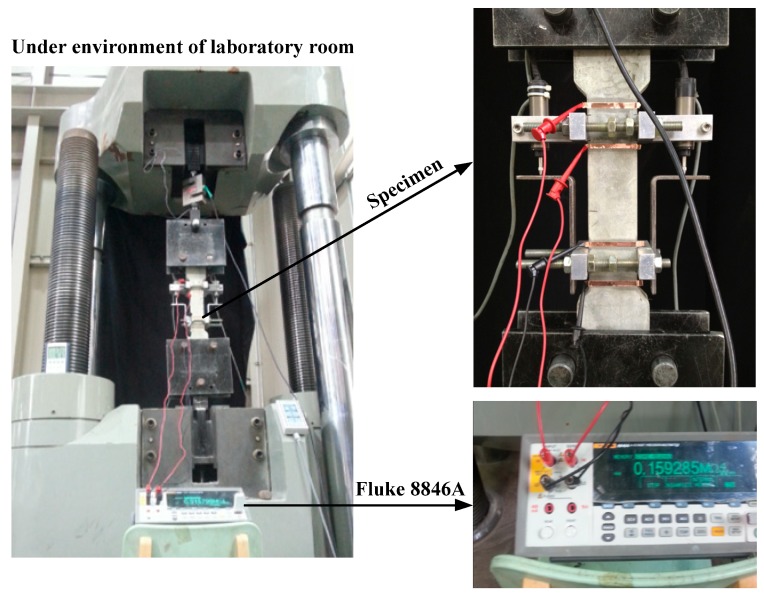
Electro-tensile test setup.

Figure 4 shows the geometry of the specimen. Bell-shaped tensile specimens were used and the section of specimens within 100 mm gauge length was 50 × 25 mm^2^, as can be seen in Figure 4. Four electrodes were installed on the surface of the specimen for measuring the electrical resistance of specimens under tension: two inner electrodes for voltage and two outer ones for current. The detail of preparing the electrodes was provided by Nguyen et al. [14]. Figure 5 shows the test setup to measure specimen resistances under controlled environmental conditions using a chamber while Figure 6 shows the electro-tensile test setup. The tensile specimens were tested under a dry state in a testing room at 25 ± 3 °C and a relative humidity of 50 ± 6%. The measured electrical resistance of the specimens can be transformed into electrical resistivity (ρ) of the material using Equation (1):

(1) $$\rho =R\cdot \frac{A}{L}$$

where *A* is the area of cross-section; *L* is the gauge length of the specimen, and also the space between the two inner electrodes; *R* is the electrical resistance and ρ is the electrical resistivity.

## 3. Results and Discussion

### 3.1. Influence of Additive Type on the Electrical Resistivity of MSFRCs

**Figure 7:**
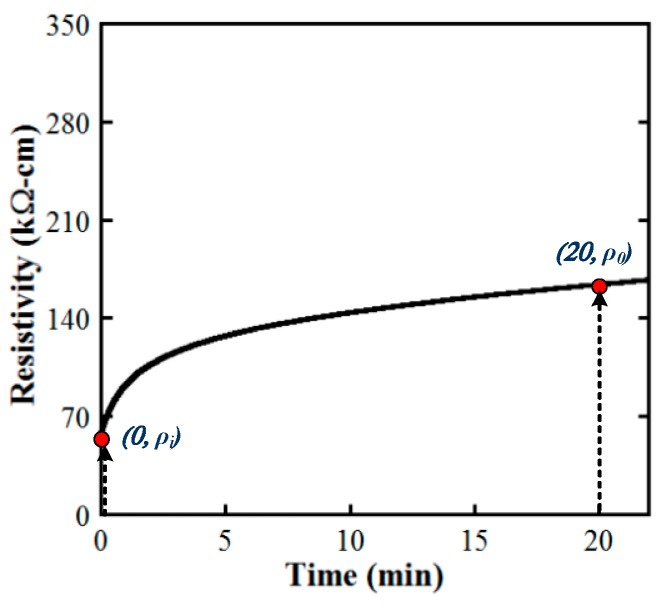
Representative electrical polarization response of cement-based composites.

Under an applied DC electrical field, the electrical resistivity of cement-based composites changes because of the polarization phenomenon [22,23,24]. As the applied DC electrical field and the polarization-induced electrical field are opposed in direction, the increase of electrical resistivity regarding time resulted from the polarization [24]. Figure 7 provides the typical electrical resistivity behavior of MSFRCs owing to the electrical polarization. As described in Figure 7, the electrical resistivity increases quickly for the first 5 min and then becomes more and more stable. In this article, the initial resistivity (ρ*_i_*) as well as the stable resistivity at 20 min (ρ_0_) of MSFRCs is compared under the same environmental condition: at 20 °C for temperature and 70% for relative humidity.

**Figure 8:**
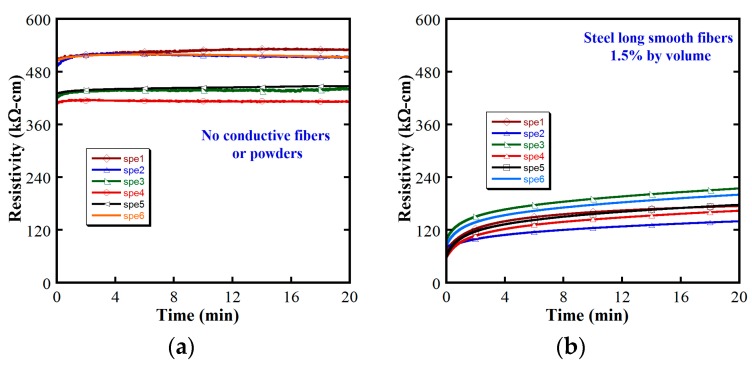

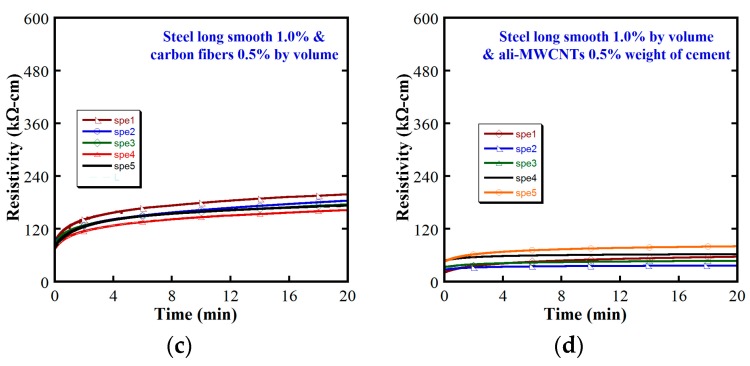
Electrical polarization responses of studied MSFRCs: (**a**) Plain mortar without fiber and powder; (**b**) 1.5 vol.% steel smooth fibers; (**c**) 1.0 vol.% steel smooth fibers & 0.5 vol.% carbon fibers; (**d**) 1.0 vol.% steel smooth fibers & 0.5% weight of cement MWCNTs.

**Figure 9:**
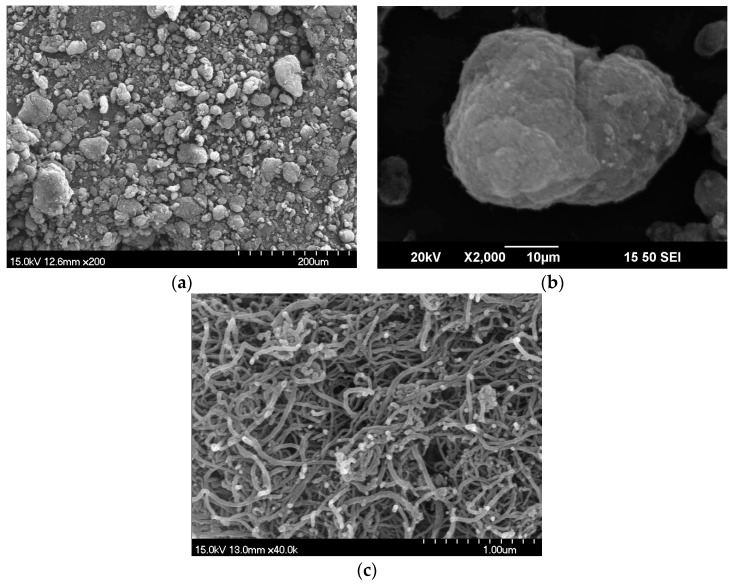
Microstructure of aligned MWCNTs under various scales by SEM: (**a**) scale 1:200; (**b**) scale 1:2000; (**c**) scale 1:40000.

The electrical resistivity behaviors of four investigated MSFRCs are shown in Figure 8. MSFRCs containing conductive materials with higher electrical conductivity or with higher volume fraction produced low electrical resistivity. The electrical resistivity response curves in all figures are generally consistent although the curves in Figure 8a are fairly dispersed. The electrical polarization is unclear as shown in Figure 8a (plain mortar) and Figure 8d (adding MWCNTs), i.e., there is little difference between ρ*_i_* and ρ_0_. The average ρ*_i_*, ρ_0_ and their ratio is provided in Table 4. The highest electrical resistivity was found in the plain mortar containing no conductive material. After adding conductive fibers or powders, the electrical resistivities of MSFRCs decreased importantly in the following order: steel smooth fibers 1.0 vol.% blended MWCNTs < hybrid fibers 1.5 vol.% < steel smooth fibers 1.5 vol.% < no conductive fibers or conductive powders. However, the ratios ρ_0_/ρ*_i_* of investigated MSFRCs were in this order: no conductive fibers nor conductive powders (1.04) < steel smooth fibers 1.0 vol.% blended MWCNTs (1.69) < hybrid fibers 1.5 vol.% (2.16) < steel smooth fibers 1.5 vol.% (2.34). Table 5 provides the normalized electrical resistivity of studied MSFRCs. As presented in Table 5, the MSFRC containing MWCNTs produced the greatest reduction in electrical resistivity, less than 7 times compared with that of plain mortar. Figure 9 shows the microstructure of aligned MWCNTs under various scales using a scanning electron microscope (SEM). The great aspect ratio and extremely small size of MWCNTs lead to the best interfacial contact with the matrix, consequently, the blend of steel fibers and MWCNTs resulted in a large reduction in electrical resistivity. It was also observed that the best conductivity of MSFRCs did certainly not produce the highest damage-sensing capability, because, under loading, the greater relative change in the electrical resistivity per unit strain is needed to generate the higher value of damage-sensing capacity.

Under applied DC current, the profile of the resistivity versus time response curve increases quickly at the start and then more and more gently over time. To describe the polarization response of the electrical resistivity versus time, a mathematical equation, ρ(*t*), for the electrical resistivity of the investigated MSFRCs corresponding to time, is suggested in this research using the inverse curve given by Equation (2).

(2) $$\rho (t)=\rho_{i-av}+\frac{t}{a+bt}$$

where ρ(*t*) is the electrical resistivity (KΩ-cm) corresponding to the time value of *t* (min.); ρ*_i_*_−*av*_ is the averaged initial electrical resistivity (KΩ-cm) corresponding to a time of zero; *a* and *b* are constants indicating material property about electrical resistivity.

**Figure 10:**
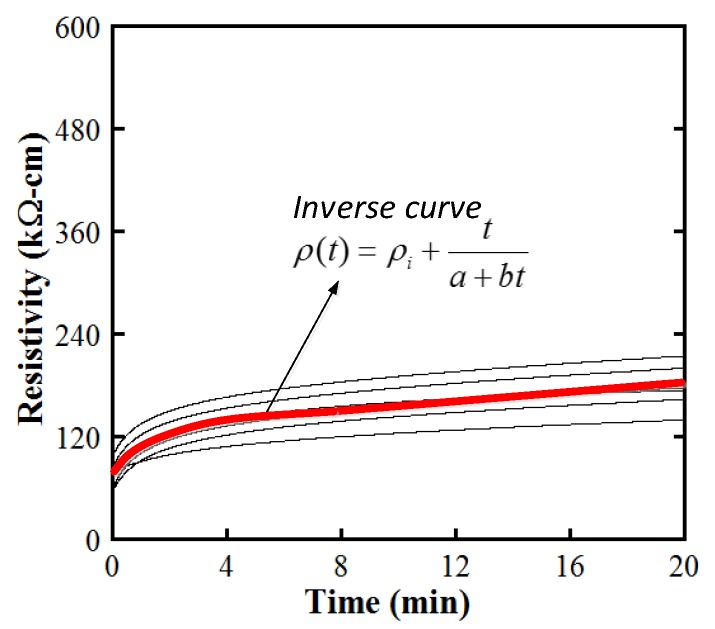
Suggested fitting curve for the polarization behavior of MSFRCs.

Figure 10 shows a suggested fitting curve for the polarization behavior of investigated MSFRCs. The coefficients *a* and *b* of the inverse curve could be drawn from regression of the testing data. The regression curve was analysed using the least-squares method and it can be used for detecting ρ(*t*) at a further time. For example, in case of MSFRC containing steel smooth fibers 1.5 vol.% (Figure 8b), the inverse curve was analytically derived as $\rho (t)=76.28+\frac{t}{0.0388+0.0089t}$.

### 3.2. Effects of Humidity and Temperature on Electrical Resistivity of MSFRC

**Figure 11:**
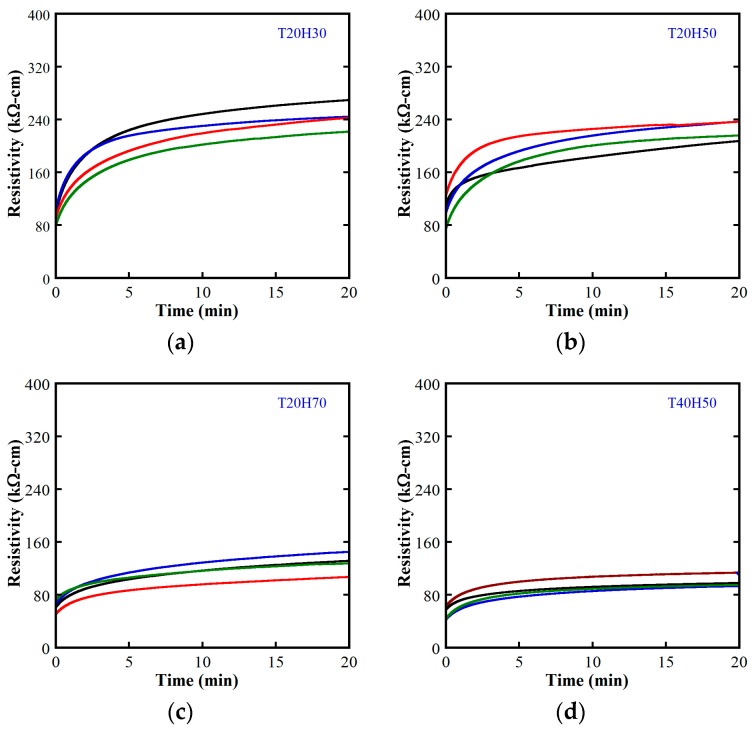
Electrical resistivity responses of MSFRC under different environmental conditions: (**a**) at temperature 20 °C, humidity 30%; (**b**) at temperature 20 °C, humidity 50%; (**c**) at temperature 20 °C, humidity 70%; (**d**) at temperature 40 °C, humidity 50%.

**Figure 12:**
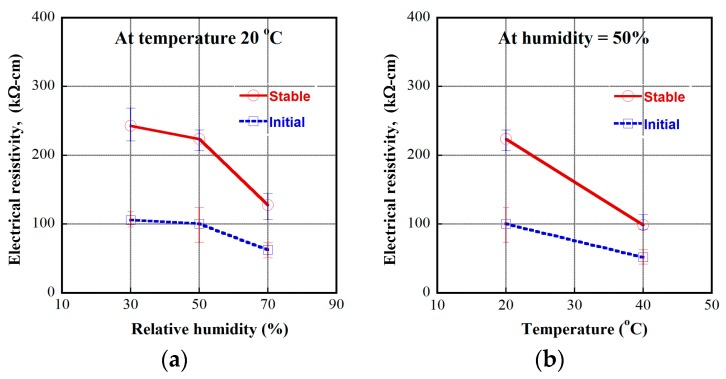
Dependence of electrical resistivity on environmental condition of MSFRC: (**a**) Effect of relative humidity; (**b**) Effect of temperature.

**Figure 13:**
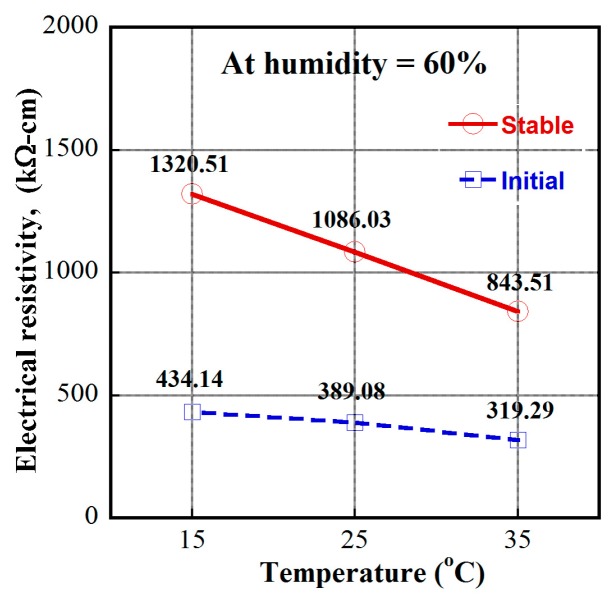
Dependence of electrical resistivity on environmental condition of UHPFRC.

In this article, the investigated MSFRCs contained 1.0 vol.% macro-steel smooth fibers blended with 1.0 vol.% carbon fibers. The electrical resistivity responses of MSFRCs varied with the environmental condition, as shown in Figure 11. The measured electrical resistivities at initial and stable time (20 min) under polarization are provided in Table 6. Figure 12a shows the effect of relative humidity on the resistivity of MSFRCs at the unchanged temperature of 20 °C, while Figure 12b displays the influence of temperature on the resistivity at the unchanged relative humidity of 50%. As shown in Figure 12a, both the initial and stable resistivity of the MSFRC significantly decrease as the relative humidity increases. Besides, when the relative humidity is more than 50%, the slope of the curves in Figure 12a is comparatively stiffer, i.e., the electrical resistivity of the MSFRC is more sensitive to the humidity. In addition, the measured resistivities clearly decrease with increasing temperature, from 20 to 40 °C, as shown in Figure 12b. The tested specimens at higher humidity have more water molecules, which cause a reduction in resistivity. In addition, the MSFRC can be considered as a semiconductor material; at high temperature, the increase of dopant atom density in the semiconductor leads to a reduction in resistivity [25]. Recently, Kim et al. [16] also explored the dependence of electrical resistivity of ultra-high performance fiber-reinforced concretes (UHPFRC) on the environment. The investigated UHPFRC used a hybrid fiber system: long smooth steel fiber 1.0 vol.% combined with short smooth steel fiber 1.0 vol.%. At a relative humidity of 60%, the electrical resistivity was examined at three temperature levels: 15 °C → 25 °C → 35 °C. Figure 13 shows the effect of temperature on electrical resistivity of UHPFRC: as the temperature increases, the electrical resistivity decreases. There were consistent results for both HPFRC and UHPFRC.

### 3.3. Effects of Carbon Fiber Volume Fraction on the Electro-Tensile Behavior of MSFRCs

**Figure 14:**
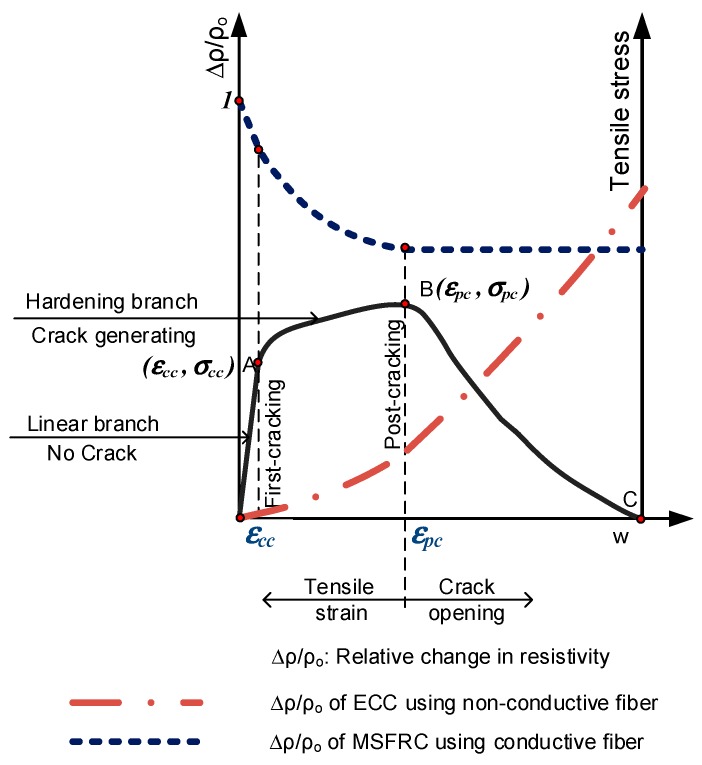
Representative electro-tensile behavior of strain-hardening composites.

The typical electro-tensile behavior of strain-hardening MSFRCs is illustrated by Figure 14. According to this figure, the strain-hardening zone of MSFRCs using conductive fiber importantly causes a reduction in electrical resistivity and damage-sensing capability of material, this mechanism was reported in detail by Nguyen et al. [14]. On the contrary, the strain-hardening zone of engineered cementitious composites (ECCs) using PVA fibers (a non-conductive material) showed an increase in electrical resistivity [18], i.e., this composite produces a reverse trend in electrical resistivity, compared with MSFRCs.

A gauge factor (GF) is a tool to evaluate the damage-sensing capability of materials and is defined as the fractional changes in the electrical resistance per unit strain. The absolute GF for a strain-hardening cement-based composite can be derived using Equation (3).

(3) $$\mathrm{GF}=\left|\frac{\Delta R/R_{0}}{\Delta \varepsilon }\right|=\left|\frac{\left(R_{0}-R_{pc}\right)/R_{0}}{\left(\varepsilon_{pc}-0\right)}\right|=\left|\frac{\left(R_{0}-R_{pc}\right)}{R_{0}\cdot \varepsilon_{pc}}\right|=\left|\frac{\left(\rho_{0}-\rho_{pc}\right)}{\rho_{0}\cdot \varepsilon_{pc}}\right|$$

where *R*_0_ (or ρ_0_) and *R*_pc_ (or ρ_pc_) are the electrical resistance (or resistivity) at the start of loading and the post-cracking point, respectively.

**Figure 15:**
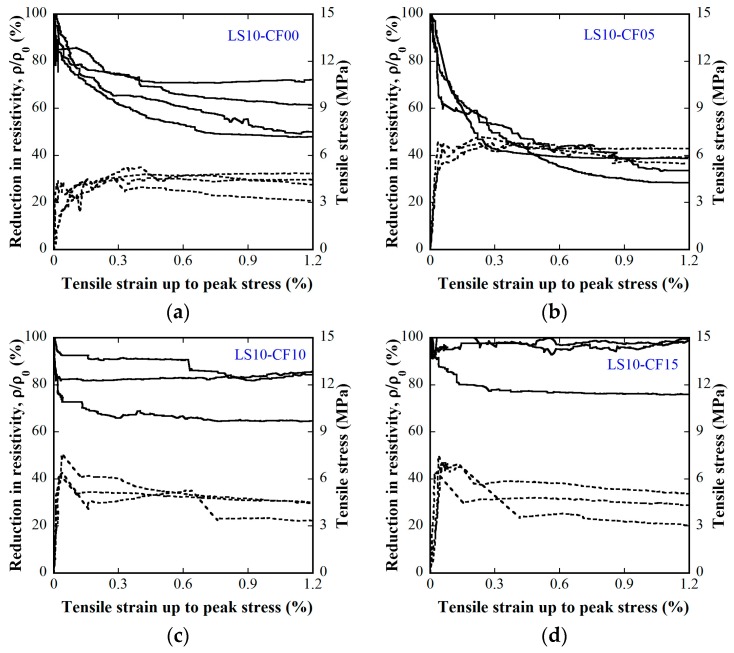
Influence of carbon fiber content on electro-tensile behaviors of MSFRC1 group (stress–strain: dashed curve, reduction in resistivity–strain: solid curve): (**a**) 1.0% smooth fibers & 0.0% carbon fibers; (**b**) 1.0% smooth fibers & 0.5% carbon fibers; (**c**) 1.0% smooth fibers & 1.0% carbon fibers; (**d**) 1.0% smooth fibers & 1.5% carbon fibers.

**Figure 16:**
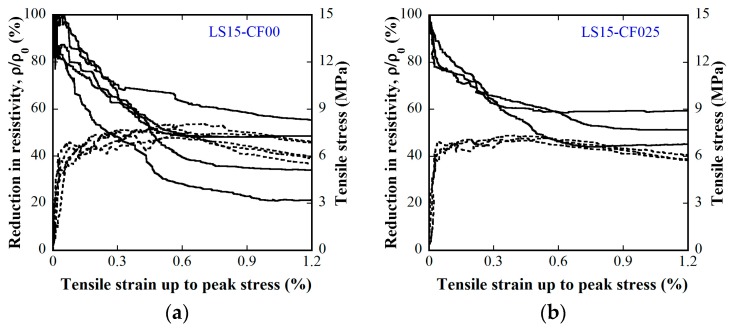

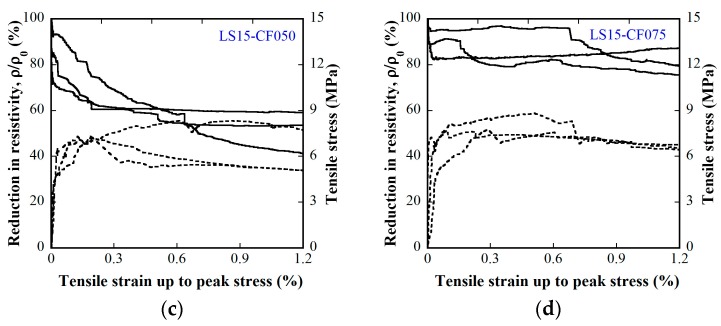
Influence of carbon fiber content on electro-tensile behaviors of the MSFRC2 group (stress–strain: dashed curve, reduction in resistivity–strain: solid curve): (**a**) 1.5% smooth fibers & carbon fibers 0.0%; (**b**) 1.5% smooth fibers & 0.25% carbon fibers; (**c**) 1.5% smooth fibers & 0.50% carbon fibers; (**d**) 1.5% smooth fibers & 0.75% carbon fibers.

**Figure 17:**
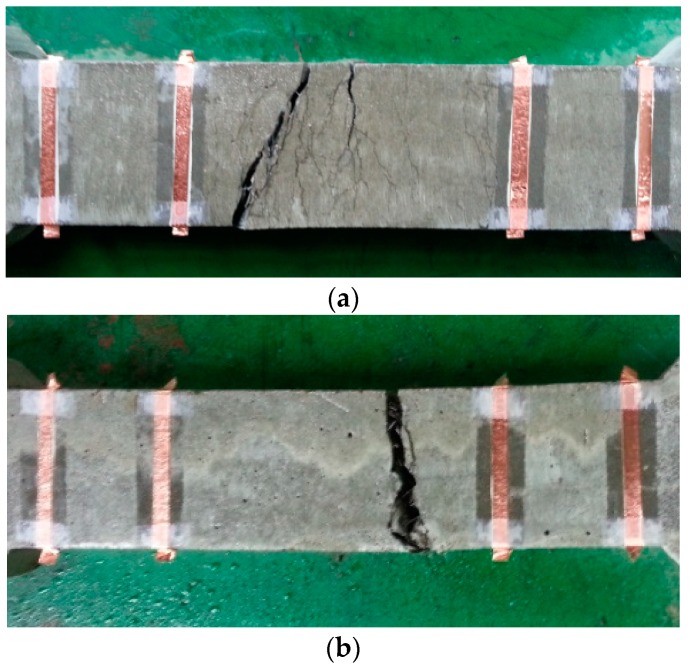
Representative cracking behavior of studied MSFRCs: (**a**) multiple tiny cracks for strain-hardening response; (**b**) single crack for strain-softening response.

**Figure 18:**
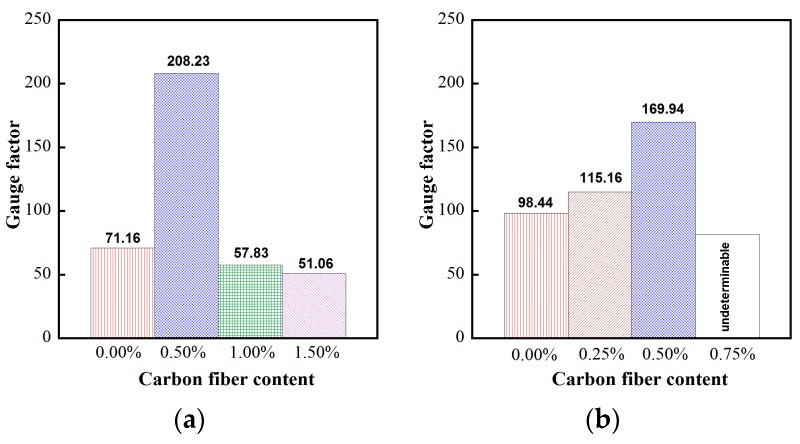
Comparative gauge factors of studied MSFRCs: (**a**) for MSFRC1 group; (**b**) for MSFRC2 group.

**Figure 19:**
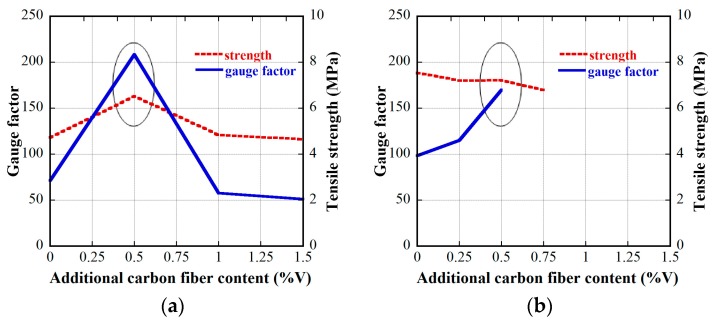
Trend of tensile strength and gauge factor of MSFRCs: (**a**) for MSFRC1 group; (**b**) for MSFRC2 group.

Figure 15 and Figure 16 provide the electro-tensile behaviors of MSFRC1 group and MSFRC2 group, respectively. In these figures, the dashed curves and the solid curves describe tensile performances and piezoresistivity performances, respectively. As shown in Figure 15, series LS1.0-CF0.0 and LS1.0-CF0.5 exhibited tensile strain-hardening behaviors accompanied by a clear reduction in resistivity, whereas series LS1.0-CF1.0 and LS1.0-CF1.5 exhibited tensile strain-softening behaviors accompanied by no clear reduction in resistivity. In addition, both the tensile and piezoresistivity response curves of series LS1.0-CF0.0 and LS1.0-CF0.5 were very consistent, whereas those of series LS1.0-CF1.0 and LS1.0-CF1.5 were not. In Figure 16, all series showed strain-hardening behaviors although distributions of their tensile response curves were differrent: the tensile curves of series LS1.5-CF0.0 and LS1.5-CF0.25 were rather consistent, whereas those of series LS1.5-CF0.50 and LS1.5-CF0.75 were scattered. Also, series LS1.5-CF0.75 shows unclear and scattered reduction in resistivity during strain-hardening. Generally, low volume content of carbon fiber added into MSFRCs would produce strain-hardening behaviors and a clear reduction in resistivity. Figure 17 shows the representative cracking behaviors of investigated MSFRCs: Figure 17a shows the strain-hardening response with multiple tiny cracks, while Figure 17b shows the strain-softening response with a single crack. The comparative gauge factors of MSFRC1 and MSFRC2 are shown in Figure 18. The optimal carbon fiber volume content to produce the highest gauge factor (208.23 for MSFRC1 and 169.94 for MSFRC2) was observed to be 0.5%. With the same additional carbon fiber (at the optimal 0.5% volume content), the gauge factor of MSFRC1 increased 2.93 times compared with no carbon fiber added (208.23/71.16-LS1.0-CF0.5/ LS1.0-CF0.0), whereas the gauge factor of MSFRC2 increased 1.73 times (169.94/98.44-LS1.5-CF0.5/ LS1.5-CF0.0). This means MSFRC1 is more sensitive to the additional carbon fibers than MSFRC2. Figure 19 provides the trend of tensile strength (dash curve) and the gauge factor (solid curve) of the investigated MSFRCs. As shown in Figure 19, LS1.0-CF0.5 is the optimal one in the MSFRC1 group, i.e., it still retained strain-hardening behavior with the highest value in both mechanical resistance and damage-sensing capacity. However, in the MSFRC2 group, the more carbon fibers added, the higher the damage-sensing capacity that was produced but the lower the tensile strength. Comparatively, LS1.5-CF0.5 is the optimal one with strain-hardening behavior, highest damage-sensing capacity and also good tensile strength. Table 7 shows the tensile resistance and fiber distribution of investigated MSFRCs. As presented in Table 7, the order of the post-cracking strength (σ_pc_**)** is as follows: LS1.0-CF0.5 > LS1.0-CF1.0 > LS1.0-CF0.0 > LS1.0-CF1.5 in group MSFRC1, and LS1.5-CF0.0 > LS1.5-CF0.5 > LS1.5-CF0.25 > LS1.5-CF0.75 in group MSFRC2. The order of strain capacity (ε_pc_**)** is not consistent with that of post-cracking tensile strength (σ_pc_**)** in both group MSFRC1 and group MSFRC2. The estimated numbers of hybrid fibers within a cross-section was computed using Equation (4) and summarized in Table 7. The number of hybrid fibers significantly depends upon the amount of carbon fibers owing to its very small diameter. The optimal numbers of hybrid fibers to produce the highest gauge factor, provided in Figure 18, was about 76,000 to 98,000 within a cross-sectional area for both LS1.0-CF0.5 and LS1.5-CF0.5, respectively.

**Figure 20:**
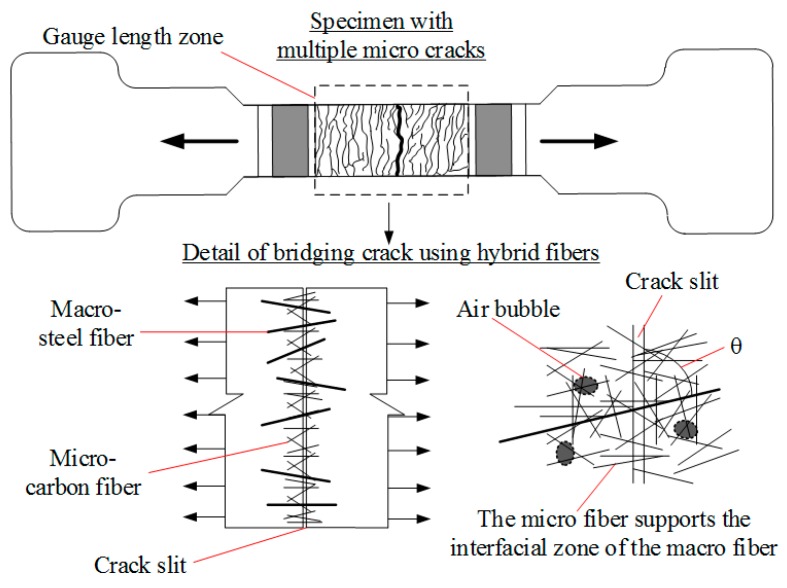
Mechanism of bridging crack using hybrid fibers.

The material properties and interfacial contacts between the fibers and the matrix could be used to explain the test results. On the one hand, compared with steel fibers, carbon fibers have higher corrosion-resistance, stiffness and tensile strength. Furthermore, carbon fibers have a very high aspect ratio and many fibers within each unit area of section, i.e., the interfacial contact between the fibers and the matrix increased and led to the reduction in electrical conductivity of the composites. On the other hand, the blend of macro-steel and micro-carbon fibers may have a large effect on the enhancement of both the damage-sensing capacity and the mechanical properties of composites, as described in Figure 20. The mechanism of bridging cracks using hybrid fibers in MSFRCs is as follows:

- In the first stage, under a low tensile load applied, the micro fibers can bridge small air bubbles and limit the crack propagation. The interfacial zone around the macro fiber is stronger with support from the micro fiber.
- In the second stage, as the crack slit through the section appears, both the micro and the macro fiber bridge the crack slit together but the macro is thought to play the main role. Park et al. [26] stated that the macro fiber mainly serves to enhance the ductility of composites while the micro fiber serves to improve the post-cracking strength.

However, with a large amount of carbon fibers added, the dispersion of carbon fibers becomes hard [27]; it is thought that there is not enough matrix bonding the fibers, which are easily tangled. This causes low interfacial contact between the fibers and the matrix leading to low tensile resistances. Moreover, MSFRC1 contains lower steel fiber content by volume than MSFRC2, thus the dispersion of hybrid fibers in MSFRC1 is more convenient, easily workable and finally leads to enhanced tensile resistance and gauge factor.

(4) $$N=\alpha_{2}\times \left(\frac{V_{f,macro}}{A_{f,macro}}+\frac{V_{f,micro}}{A_{f,micro}}\right)\times A_{c}$$

where *N* is the fiber number within a cross-section of the specimen, *A*_c_ (mm^2^) α_2_ is the coefficient of fiber orientation, which simply equals 1 for 1D distribution; α_2_ for 2D and 3D are equal to 2/π, and 0.5 using Equations (5) and (6), respectively.

(5) $$\alpha_{2-2D}=\int_{0}^{\pi /2}\frac{\sin\theta }{\pi /2}d\theta =\frac{2}{\pi }$$

(6) $$\alpha_{2-3D}=\int_{0}^{\pi /2}\sin\theta \cos\theta d\theta =1/2$$

where *V_f,macro_* and *A_f,macro_* are the content by volume and section area of macro fibers, respectively, while *V_f,micro_* and *A_f,micro_* are those of micro fibers.

### 3.4. Damage-Sensing Capabilities of Some MSFRCs in Review

**Figure 21:**
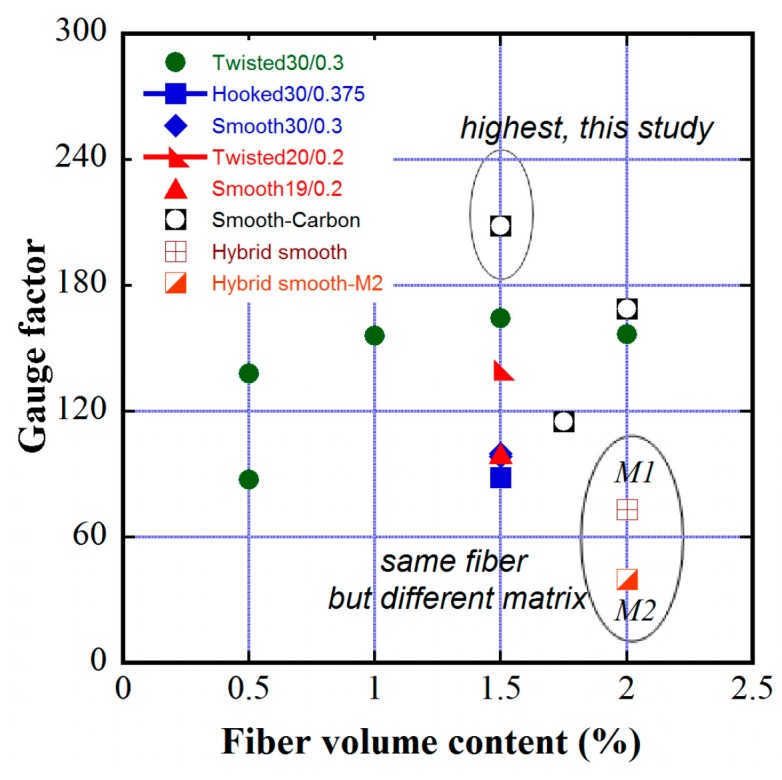
Distribution of gauge factor of MSFRCs regarding fiber type and content.

Table 8 summarizes the damage-sensing capacities of strain-hardening composites by means of their GFs, in addition to the post-cracking tensile strengths. As shown in Table 8, the GFs are greatly dependent upon fiber types, fiber volume fraction and matrix properties. The matrix M2 (No.15) produces the highest compressive strength and highest post-cracking strength but lower GF than matrix M1 (No.14), although both matrices contain the same fiber type and fiber content. The MSFRCs that use twisted fibers (e.g., No.1, No.7, No.08) generally produce high GF and high post-cracking tensile strength. Figure 21 shows the GF distribution of MSFRCs regarding fiber type and content. Remarkably, MSFRCs using 1% smooth fiber blended 0.5% carbon fiber by volume (No.10) produce the highest GF, but do not produce high tensile strength. The hybrid system of long smooth fiber and low carbon fiber produced a favorable effect by enhancing the damage-sensing capacity of MSFRCs.

### 3.5. Effects of MWCNTs on the Electro-Tensile Behavior of MSFRCs

It has been hard to disperse MWCNTs because it has a high tendency to cluster, owing to van der Waals forces [28]. In this research, MWCNTs were dispersed by an ultrasonicator with three cases of mixtures as follows: (a) water, superplasticizer, NaDDBS (sodium sodium dodecylbenzene sulfonate); (b) water, superplasticizer, NaDDBS and tributyl phosphate; (c) water, superplasticizer. The NaDDBS was used as a surfactant, while the tributyl phosphate performed as a defoamer to limit the voids caused by NaDDBS (refer to Han et al. [19]). The MWCNTs were observed to absorb much water, as shown in Figure 3, thus more water was added in each case by mixing to obtain suitable workability and viscosity.

**Figure 22:**
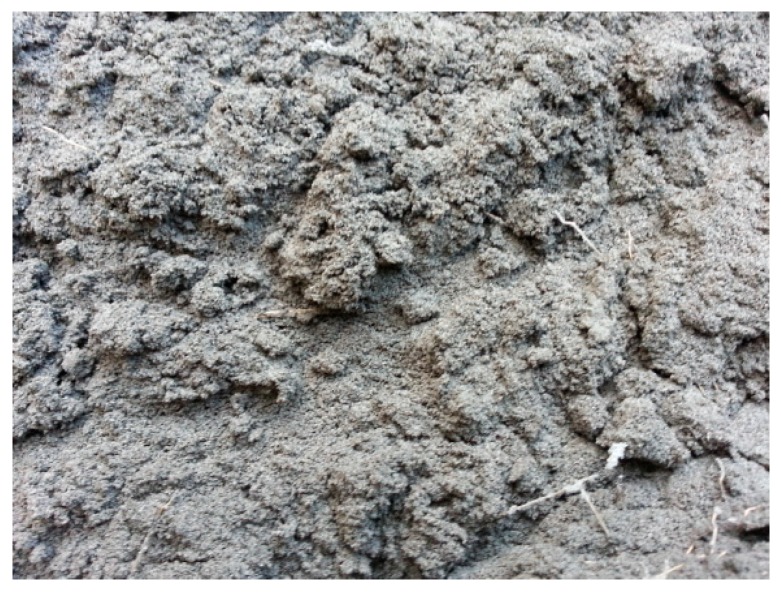
Foam concrete generated by NaDDBS.

**Figure 23:**
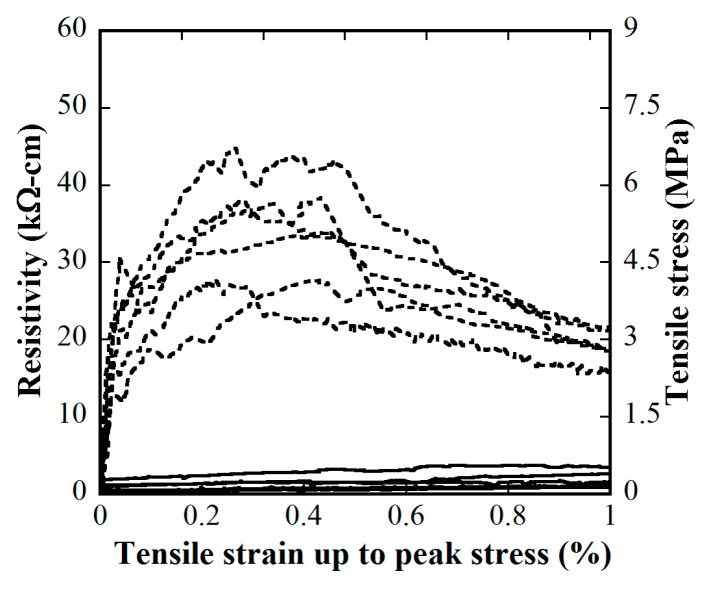
Electro-tensile behaviors of MSFRC2 containing MWCNTs using water, superplasticizer, NaDDBS and tributyl phosphate.

**Figure 24:**
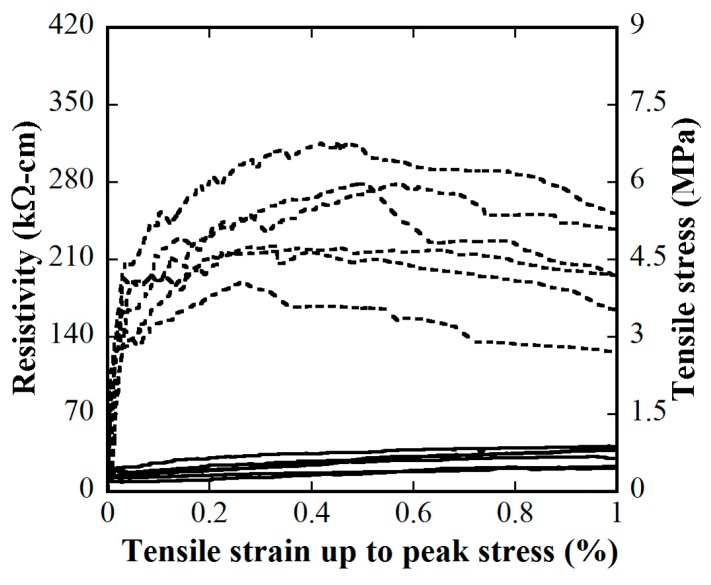
Electro-tensile behaviors of MSFRC2 containing MWCNTs using only water and superplasticizer.

The MSFRC2s containing 0.5% MWCNTs exhibited considerably different behaviors according to the mentioned cases. In the case of (a), MSFRC2 was very spongy, i.e., the product gets foamed as shown in Figure 22, consequently, its tensile resistance was nearly omitted. In the case of (b), the MSFRC2 did not foam owing to the tributyl phosphate effect; the electro-tensile behavior of MSFRC2 is shown in Figure 23. As shown in Figure 23, the tensile stress versus strain response curves of MSFRC2 were importantly scattered, while its electrical resistivity versus strain response curves were rather gathered with a positive correlation (inverse trend compared with Figure 14). There was no enhancement of tensile resistance of MSFRC2 in this case, compared with the original described in Figure 16a. The average post-cracking strength was 5.25 MPa, whereas that of the original was 7.53 MPa. Furthermore, the measured electrical resistivity in this case was significantly reduced with an average value of 1.26 kΩ-cm at the peak stress. In the case of (c), the MSFRC2 did also not foam. Figure 24 shows the electro-tensile behavior of MSFRC2; the tensile stress versus strain response curves were scattered with an average post-cracking tensile strength of 5.38 MPa only; the electrical resistivity versus strain exhibited a positive correlation (inverse trend compared with Figure 14). The average value of resistivity at peak stress was 24.14 kΩ-cm. In both cases of (b) and (c), the GFs were not enhanced because their relative changes in resistivity were low; multiple micro cracking behaviors occured but the numbers of cracks were counted from 2 to 5 only. Although the enhancement of tensile resistance and damage-sensing capacity were not successful as expected, the testing showed the true difficulty in producing MSFRCs reinforced by MWCNTs, and, further test programs with new methods are needed.

## 4. Conclusions

An attempt to enhance the damage-sensing capacity of MSFRCs was experimentally conducted in this study. Although this study provides some useful information in developing self-sensing construction materials, further investigation is required to deeply understand the electro-mechanical damage-sensing mechanism of MSFRCs. These following conclusions can be drawn from the test results:

- From same plain mortar, adding conductive additives clearly affected the electrical resistivity of MSFRCs. The order of studied MSFRCs in term of the electrical resistivity was ranked as follows: steel smooth fibers 1.0% volume content blended MWCNTs < hybrid fibers 1.5% volume content < steel smooth fibers 1.5% volume content < no conductive fibers or conductive powders. This means that the MWCNTs strongly enhanced the electrical conductivity of MSFRCs.
- The low electrical resistivity of the MSFRCs was not accompanied by a high damage-sensing capacity that is strongly dependent on the relative change in resistivity.
- The environmental condition clearly affected the electrical resistivity of MSFRCs; as the relative humidity or temperature increased, there was an important reduction in electrical resistivity of MSFRCs.
- Adding 0.5% volume fraction of carbon fibers in initial MSFRC1 or MSFRC2, containing 1.0% and 1.5% volume content of long smooth fibers, respectively, produced the highest gauge factor, i.e., the highest damage-sensing capability. Furthermore, MSFRC1 was more sensitive to the additional carbon fiber than MSFRC2.
- The high volume content of carbon fibers added may cause the reduction in gauge factor, specifically, the carbon fibers, in a hybrid system with steel fibers, should not exceed 0.5% volume fraction in order to disperse well and produce strain-hardening. The post-cracking resistances of MSFRC1 generally increased with addition of low volume content of carbon fibers, whereas those of MSFRC2 did not.
- Although ultra-high-performance concrete (matrix M2) can produce very high compressive strength as well as the highest post-cracking tensile strength, it might not produce a high gauge factor.
- Adding MWCNTs into original MSFRCs, with their fabrications described in this study, might not produce a favorable enhancement of mechanical resistance as well as damage-sensing capacity of MSFRCs, although their electrical resistivity would be significantly reduced.

## Figures and Tables

**Table 1:** Composition of mortar matrix and its compressive strength.

Cement (Type 3)	Silica Fume	Silica Sand	Fly Ash	Superplasticizer	Water	f_c_’ (MPa)
0.80	0.07	1.00	0.20	0.04	0.26	90

The value of composition was described using weight ratio.

**Table 2:** Properties of macro-steel smooth fibers and micro-carbon fibers.

Parameters	Micro-carbon Fiber	Macro-steel Smooth Fiber Fiber
Product origin	Panex35 (Korea)	JKT (Korea)
Diameter (mm)	0.0072	0.3
Length (mm)	12	30
Aspect ratio (Length/Diameter)	1667	100
Density (g/cc)	1.81	7.9
Tensile strength (MPa)	4137	2580
Elastic modulus (GPa)	240	200
Electrical resistivity (kΩ-cm)	1.2	2.06 × 10^−8^

**Table 3:** Properties of MWCNTs.

Parameters	Value
Product origin	Hanwha (Korea)
Purity (wt.%)	>90
Bulk density (g/cc)	0.065
Aspect ratio	2000
Diameter (nm)	10~15
Length (μm)	30~40
Number of wall	10~15
Ash (catalyst residue, %)	10%
Surface area (m^2^/g)	~1200

**Table 4:** Electrical resistivity of studied MSFRCs regarding various conductive additives.

Type of MSFRC	ρ*_i_* (kΩ-cm)		ρ_0_ (kΩ-cm)		Ratio ρ_0_/ρ*_i_*
	Average Value	Standard Deviation	Average Value	Standard Deviation	
Plain mortar without fiber and powder	458.60	44.83	475.84	48.99	1.04
Steel smooth 1.5 vol.%	76.28	16.70	178.15	26.48	2.34
Steel smooth 1.0% & CF 0.5 vol.%	82.06	6.68	177.36	12.45	2.16
Steel smooth 1.0 vol.% & MWCNTs 0.5 wt.% of cement	39.10	11.73	65.99	15.57	1.69

**Table 5:** Normalization of electrical resistivity of studied MSFRCs.

Type of MSFRC	Normalized, ρ*_i_*	Normalized, ρ_0_
Plain mortar without fiber & powder	1	1
Steel smooth 1.5 vol.%	0.17	0.37
Steel smooth 1.0% & CF 0.5 vol.%	0.18	0.37
Steel smooth 1.0 vol.% & MWCNTs 0.5 wt.% of cement	0.09	0.14

**Table 6:** Electrical resistivity of studied MSFRCs regarding temperature and humidity.

Environmental Condition	ρ*_i_* (kΩ-cm)		ρ_0_ (kΩ-cm)	
	Average Value	Standard Deviation	Average Value	Standard Deviation
Temperature 20 °C, humidity 30%	105	10.6	243	19.7
Temperature 20 °C, humidity 50%	101	21.4	224	14.0
Temperature 20 °C, humidity 70%	63	9.3	128	15.7
Temperature 40 °C, humidity 50%	52	10.3	99	10.2

**Table 7:** Post-cracking tensile resistance and fiber distribution of MSFRCs.

Notation	Post-Cracking Parameters		Estimated Number of Hybrid Fibers within Cross-Section	
	σ_pc_ (MPa)	ε_pc_ (%)	*N*_2D_	*N*_3D_
LS1.0-CF0.0	4.73	0.42	113	88
LS1.0-CF0.5	6.51	0.45	97837	76841
LS1.0-CF1.0	4.82	0.28	195562	153594
LS1.0-CF1.5	4.64	0.35	293287	230347
LS1.5-CF0.0	7.53	0.44	169	133
LS1.5-CF0.25	7.19	0.37	49031	38509
LS1.5-CF0.5	7.21	0.22	97894	76886
LS1.5-CF0.75	6.79	0.29	146756	115262

**Table 8:** Gauge factors of strain-hardening composites using various conductive discrete fiber.

No.	Fiber used: Type, Aspect Ratio (mm/mm), Volume Content [ref.]	GF	Matrix	Post-Cracking Strength (MPa)
01	Twisted, 30/0.3, 1.5% [14]	138.09	M1	10.00
02	Smooth, 30/0.3, 1.5% [14]	99.85	M1	7.64
03	Hooked, 30/0.375, 1.5% [14]	88.50	M1	6.72
04	Twisted, 20/0.2, 1.5% [14]	139.68	M1	10.99
05	Smooth, 19/0.2, 1.5% [14]	99.70	M1	8.05
06	Twisted, 30/0.3, 0.5% [15]	87.26	M1	4.86
07	Twisted, 30/0.3, 1.0% [15]	155.99	M1	7.48
08	Twisted, 30/0.3, 1.5% [15]	164.24	M1	9.99
09	Twisted, 30/0.3, 2.0% [15]	156.54	M1	12.53
10	Smooth, 30/0.3, 1.0% & Carbon, 12/0.0072, 0.5% [this study]	208.23	M1	6.51
11	Smooth, 30/0.3, 1.5% [this study]	98.44	M1	7.53
12	Smooth, 30/0.3, 1.5% & Carbon, 12/0.0072, 0.25% [this study]	115.16	M1	7.19
13	Smooth, 30/0.3, 1.5% & Carbon, 12/0.0072, 0.5% [this study]	169.94	M1	7.21
14	Smooth, 30/0.3, 1.0% & Smooth, 19/0.2, 1.0% [16]	73.22	M1 (*)	12.37
15	Smooth, 30/0.3, 1.0% & Smooth, 19/0.2, 1.0% [16]	39.88	M2 (**)	15.13

(*) M1 is the matrix of high-performance concrete, composition of M1 see Table 1. (**) M2 is the matrix of ultra-high-performance concrete with compressive strength of 180 MPa, composition of M2 [16].
